# How Charge, Size and Protein Corona Modulate the Specific Activity of Nanostructured Lipid Carriers (NLC) against *Helicobacter pylori*

**DOI:** 10.3390/pharmaceutics14122745

**Published:** 2022-12-08

**Authors:** Rute Chitas, Cláudia Nunes, Salette Reis, Paula Parreira, Maria Cristina L. Martins

**Affiliations:** 1 i3S-Instituto de Investigação e Inovação em Saúde, Universidade do Porto, 4200-135 Porto, Portugal; 2 INEB-Instituto de Engenharia Biomédica, Universidade do Porto, 4200-135 Porto, Portugal; 3 ICBAS-Instituto de Ciências Biomédicas Abel Salazar, Universidade do Porto, 4050-313 Porto, Portugal; 4 LAQV-REQUIMTE, Departamento de Ciências Químicas, Faculdade de Farmácia, Universidade do Porto, 4050-313 Porto, Portugal

**Keywords:** antimicrobial, *H. pylori*, nanoparticles, nanostructured lipid carriers, NLC, gut microbiota, protein corona

## Abstract

The major risk factor associated with the development of gastric cancer is chronic infection with *Helicobacter pylori*. The available treatments, based on a cocktail of antibiotics, fail in up to 40% of patients and disrupt their gut microbiota. The potential of blank nanostructured lipid carriers (NLC) for *H. pylori* eradication was previously demonstrated by us. However, the effect of NLC charge, size and protein corona on *H. pylori*-specific bactericidal activity herein studied was unknown at that time. All developed NLC formulations proved bactericidal against *H. pylori*. Although cationic NLC had 10-fold higher bactericidal activity than anionic NLC, they lacked specificity, since *Lactobacillus acidophilus* was also affected. Anionic NLC achieved complete clearance in both *H. pylori* morphologies (rod- and coccoid-shape) by inducing alterations in bacteria membranes and the cytoplasm, as visualized by transmission electron microscopy (TEM). The presence of an NLC protein corona, composed of 93% albumin, was confirmed by mass spectrometry. This protein corona delayed the bactericidal activity of anionic NLC against *H. pylori* and hindered NLC activity against *Escherichia coli*. Overall, these results sustain the use of NLC as a promising antibiotic-free strategy targeting *H. pylori*.

## 1. Introduction

Since the rediscovery of *Helicobacter pylori* in 1982 by Marshall and Warren, it is known that this bacterium colonizes the human stomach and is responsible for several gastric disorders [1,2,3]. It is now estimated that *H. pylori* colonizes around half of the world population and is responsible for 90% of the gastric cancer burden [4,5], its eradication is recommended for all infected patients, even if asymptomatic [6]. Although 40 years have passed, the therapeutic regimen recommended for *H. pylori* eradication still relies on a combination of antibiotics [6,7]. However, antibiotic treatments are progressively failing (20–40%), mainly due to the increase in antibiotic resistance, but also fueled by low patient compliance to the complex treatment schemes with adverse side effects [6,8,9]. Antibiotic therapy also affects gut microbiota, causing dysbiosis, which increases susceptibility to infections by opportunistic bacteria (such as *Clostridium difficile*) and the development of inflammatory and autoimmune disorders [6,7,10].

It was recently reported that *H. pylori* can form biofilms in the stomach, where bacteria are protected by a complex biofilm matrix [11,12]. Moreover, in adverse conditions and due to its morphological plasticity, *H. pylori* can enter a low metabolically active state (“viable but non-culturable” (VBNC)), by changing its morphology from rod- to coccoid-shaped [13,14,15]. *H. pylori* in biofilms and coccoid morphology are more resistant to antibiotics, boosting the failure of conventional treatments and promoting recurrent and chronic *H. pylori* infection [14,16,17,18].

Thus, it is urgent to find an antibiotic-free alternative therapy that enables *H. pylori* eradication without promoting bacterial resistance or alteration of gut microbiota [6].

It was previously demonstrated by our group that nanostructured lipid carriers (NLC), without any drug (blank NLC), were bactericidal against *H. pylori* J99 (highly pathogenic human strain) [19,20]. These blank NLC killed *H. pylori* by altering the bacterial membrane, which led to the leakage of cytoplasmic content. This effect was not expected since this type of lipid nanoparticles, designed to increase drug loading capacity and stability [21,22,23], were only composed of generally recognized as safe (GRAS) components, namely, a mixture of biocompatible and biodegradable lipids (Precirol ATO5^®^ and Miglyol-812^®^) and a surfactant (Tween 60^®^). Additionally, these non-cytotoxic NLC did not affect other bacteria from gut microbiota [19,20]. More recently, it was also demonstrated that these blank NLC were effective against *H. pylori* biofilms [24]. These earlier results point to NLC as promising for the development of a new and safe treatment for *H. pylori*. However, the influence of NLC physicochemical parameters and protein corona on their specific antibacterial effect against *H. pylori* has yet to be an object of study. Additionally, NLC bactericidal activity against the more resistant coccoid-shaped *H. pylori* is not known. The main objective of this current work was to study the influence of NLC charge, size, and protein corona on their specific antibacterial effect against rod- and coccoid-shaped *H. pylori.* For that, NLC with different surface charges and sizes were obtained by using different surfactants (Tween^®^60 or Tween^®^80 and Cetyltrimethylammonium bromide—CTAB), and sonication parameters (time and amplitude), respectively. The different NLC formulations were tested against *H. pylori* and bacteria representative of gut microbiota, *Lactobacillus acidophilus* and *Escherichia coli*, to assess their selectivity towards *H. pylori*. NLC performance against *H. pylori* in coccoid morphology was also studied. Finally, since *H. pylori* is a fastidious bacterium and all the in vitro assays were performed in the presence of Fetal Bovine Serum (FBS), the NLC protein corona was characterized, and its effect on NLC activity and selectivity was further explored.

## 2. Materials and Methods

### 2.1. Nanostructured Lipid Carriers (NLC)

#### 2.1.1. NLC Production and Optimization

NLC were produced by hot homogenization and ultrasonication (adapted from Seabra et al.) [19]. NLC were composed of a solid lipid (Precirol^®^ATO5; Gattefossé, Saint-Priest, France), a liquid lipid (Miglyol^®^812; Acofarma, Madrid, Spain) and one of the following surfactants: (i) Tween^®^60 (NLC60); (ii) Tween^®^80 (NLC80); and (iii) different ratios of the surfactants Tween^®^60 and Cetyltrimethylammonium bromide—CTAB (NLC CTAB) in concentrations detailed in Table 1. Tween^®^60 and Tween^®^80 were obtained from Merck, Germany and CTAB from Sigma-Aldrich, St. Louis, MO, USA.

All the components were weighted and melted together at 65 °C. Then, Type I water (ultrapure water with resistivity > 18 MΩ-cm and a conductivity < 0.056 µS/cm, Milli-Q^®^, Millipore, Burlington, MA, USA), at the same temperature, was added to the blended components. Afterward, the mixture was sonicated (Vibra-Cell model VCX 130, Sonics and Materials Inc., Newtown, CT, USA) with a ∅ 6 mm tip. To obtain NLC with different sizes, the sonication time was varied from 5 min up to 20 min and the amplitude from 40% up to 90% (the tested conditions are described in Table S1).

#### 2.1.2. NLC Characterization

NLC hydrodynamic diameter and surface charge (ζ-potential) were determined by dynamic light scattering (DLS) and electrophoretic light scattering (ELS), respectively, using a Malvern Zetasizer Nano ZS (Malvern Panalytical, Malvern, UK). For sample measurement, NLC were diluted (1:50) in Type I water (Milli-Q^®^) and placed on a disposable capillary cell (DTS1070, Malvern, UK). Measurements were done at 37 °C with a backscattering angle of 173°. Values were obtained by calculating the average of three runs (each with twelve cycles). NLC concentration was assessed by nanoparticle tracking analysis (NTA) using a NanoSight^®^ NS300 (Malvern Panalytical, Malvern, UK). The samples were diluted (1:200,000) in Type I water (Milli-Q^®^) and measurements were performed in triplicate. Additionally, NLC morphology and size were evaluated by transmission electron microscopy (TEM). For this, NLC were diluted (1:200) and 10 µL of each sample were mounted on Formvar/carbon film-coated mesh nickel grids (Electron Microscopy Sciences, Hatfield, PA, USA). After 2 min, the excess liquid was removed with filter paper. For negative staining, 2 µL of 1% uranyl acetate was added to the grids. After a short incubation (10 s), excess liquid was removed with filter paper. Visualization was carried out on a JEOL 100CXII (Tokyo, Japan) at 60 kV.

### 2.2. NLC Activity against H. pylori

#### 2.2.1. Bacterial Growth Conditions

NLC were tested against the human-isolated *H. pylori* strain J99 (provided by the Department of Medical Biochemistry and Biophysics, Umeå University, Sweden) according to Seabra et al. [20]. Briefly, the bacterium was cultured in spots in specific *H. pylori* medium plates composed of blood agar base 2 (Oxoid, France) supplemented with 10% *v*/*v* defibrinated horse blood (Probiológica, Lisbon, Portugal) and 0.2% *v*/*v* of an antibiotic cocktail composed by Polymyxin B, Vancomycin, Amphotericin B and Trimethroprim (all from Sigma-Aldrich, St. Louis, MO, USA) [19]. The bacterium was grown in a microaerophilic environment (GenBox system, BioMérieux, Marcy-l’Étoile, France) for 48 h at 37 °C. Then, colonies were streaked in fresh medium plates and incubated for another 48 h under the same conditions as above. Finally, the bacteria were harvested from the solid medium, centrifuged 774× *g*, RT, 10 min (Eppendorf™ 5810R, Eppendorf, Germany) and resuspended in Müeller-Hinton broth medium (MHB, Merck, Germany) supplemented with 10% of heat-inactivated (30 min, 65 °C) Fetal Bovine Serum (FBS, Gibco, Waltham, MA, USA), prior to transfer to T-flasks with the same medium. Optical density (OD) was adjusted to 0.1 (λ = 600 nm; UV/VIS spectrophotometer, Lambda 45, Perkin Elmer, Waltham, MA, USA). To obtain rod-shaped *H. pylori*, incubation proceeded overnight (16–18 h) under microaerophilic conditions, at 37 °C and 150 revolutions per minute (rpm). For inducing *H. pylori* morphological conversion to coccoid-shaped, incubation in liquid media was done for 72 h [25].

#### 2.2.2. NLC Antibacterial Performance

After incubation in liquid medium (as described in Section 2.2.1) for 16–18 h, the bacterial culture was adjusted to approximately 1 × 10^7^ (OD ≈ 0.03) colony forming units (CFU)/mL in MHB + 10% FBS medium [26]. Different NLC concentrations (10^10^, 10^11^, 10^12^, 10^13^ particles/mL) were incubated with the bacteria over 24 h, at 37 °C in microaerophilic conditions. After 24 h, 100 µL of each sample was collected, serially diluted 10-fold in phosphate-buffered saline (PBS pH = 7.4, Sigma-Aldrich, St. Louis, MO, USA), and 20 µL of each dilution was plated in triplicate in blood agar base plates.

To compare the NLC60 effect against different *H. pylori* morphologies, two cultures were prepared: one was incubated in liquid medium for 16–18 h and another for 72 h (as described in Section 2.2.1). Afterward, cultures were centrifuged (774× *g*, RT, 10 min) and the pellets were re-suspended in PBS, being then adjusted to approximately 1 × 10^7^ CFU/mL (OD ≈ 0.03). Different NLC concentrations (10^12^ and 10^13^ particles/mL) were incubated with the bacteria for 6 h at 37 °C in microaerophilic conditions. Since this assay was performed in PBS, the time of incubation was shorter (6 h) to avoid bacterial death due to prolonged exposure to nutrient starvation (incubation in PBS). After 6 h, samples were plated as described above and incubated at 37 °C for 4–5 days in microaerophilic conditions. The number of viable bacteria/mL was calculated by CFU counting (CFU/mL = (nº colonies × dilution factor)/volume of culture plated). The NLC formulation was considered bactericidal if a reduction of at least 99.9% (≥3 log^10^) of the total count of CFU/mL was observed when compared with the control (bacteria without NLC). Each condition was tested in triplicate.

#### 2.2.3. Transmission Electron Microscopy (TEM)

*H. pylori* cultures were prepared as described in Section 2.2.1. Bacterial cultures of 16–18 h (rod) and 72 h (coccoid) incubation were centrifuged (774× *g*, RT, 10 min) and pellets were re-suspended in PBS and adjusted to approximately 1 × 10^7^ CFU/mL (OD ≈ 0.03). Two NLC60 concentrations (10^12^ and 10^13^ particles/mL) were incubated with the bacteria for 6 h at 37 °C in microaerophilic conditions. To ensure enough bacterial content for posterior sample preparation, 10 replicates of each condition were prepared. Then, all the replicates per each condition were combined and centrifuged (3000× *g*, RT, 10 min). The bacterial pellets were fixed with a solution of 2% glutaraldehyde, 2.5% formaldehyde (both from Electron Microscopy Sciences, Hatfield, PA, USA) and 0.1 M sodium cacodylate buffer (pH 7.4) for 1 h at room temperature. Then, samples were post-fixed with 1% osmium tetroxide (Electron Microscopy Sciences, Hatfield, PA, USA) diluted in 0.1 M sodium cacodylate buffer and re-suspended in Histogel^TM^ (HG-4000-012, Thermo Fisher Scientific, Bremen, Germany). Afterward, they were stained with aqueous 1% uranyl acetate solution overnight, dehydrated with ethanol and propylene oxide and then embedded in Embed- 812 resin (Electron Microscopy Sciences, Hatfield, PA, USA). Each resin was cut in ultra-thin sections of 50 nm thickness on an RMC Ultramicrotome (PowerTome, Moses Lake, WA, USA) using Diatome diamond knives (Delaware Diamond Knives, Wilmington, DE, USA) and mounted on mesh nickel grids (Electron Microscopy Sciences, Hatfield, PA, USA). Next, the sections were stained with uranyl acetate substitute and lead citrate (both from Electron Microscopy Sciences, Hatfield, PA, USA) for 5 min each. Ultra-thin sections were analyzed in a JEOL JEM 1400 transmission electron microscope (JEOL, Tokyo, Japan) and images were digitally recorded using a CCD digital camera Orius 1100W (Gatan, Tokyo, Japan).

### 2.3. NLC Activity against Gut Bacteria

#### 2.3.1. Bacterial Growth Conditions

NLC were also tested against *Escherichia coli* ATCC^®^25922™ and *Lactobacillus acidophilus*-01 strain (provided by Chr. Hansen, Hørsholm, Denmark).

*E. coli* ATCC^®^25922™ was streaked in Tryptic Soy Agar (TSA, Merck, Germany) plates and incubated at 37 °C overnight (16–18 h). Afterward, 1–2 colonies were picked and incubated in Tryptic Soy Broth (TSB, Merck, Germany) overnight under aerobic conditions at 37 °C and 150 rpm. For *L. acidophilus*-01, the bacterium was streaked in De Man, Rogosa and Sharpe agar (MRS, Biokar Diagnostics, Allonne, France) plates and incubated in microaerophilic conditions for 48 h at 37 °C. Then, 1–2 colonies were picked and incubated in De Man, Rogosa and Sharpe broth (Biokar Diagnostics, Allonne, France) overnight (16–18 h) under microaerophilic conditions at 37 °C and 150 rpm.

#### 2.3.2. NLC Antibacterial Performance

The bacterial cultures (prepared as described in Section 2.3.1) were centrifuged at 774× *g*, RT for 10 min. After, the supernatants were discarded, and the bacterial pellets were re-suspended in either MHB or MHB + 10% FBS medium. Bacterial concentration was adjusted to approximately 1 × 10^5^ CFU/mL [27]. Different NLC concentrations (10^10^, 10^11^, 10^12^, 10^13^ particles/mL) were incubated with the bacteria for 24 h at 37 °C (in microaerophilic conditions for *L. acidophilus*-01). Then, 100 µL of each sample was collected, and serially diluted 10-fold in PBS, and 10 µL of each dilution was plated in the appropriate medium plates. The plates were incubated for 48 h at 37 °C for *L. acidophilus*-01 and overnight (16–18 h) for *E. coli* ATCC^®^25922™. The number of bacteria/mL was calculated by CFU counting. Each condition was done in triplicate.

### 2.4. NLC Protein Corona

#### 2.4.1. NLC Protein Corona Characterization

NLC protein corona was analyzed by liquid chromatography with tandem mass spectrometry (LC-MS/MS). To analyze the NLC protein corona, the nanoparticles (NLC60) were incubated in different media: PBS, MHB and MHB + 10% FBS in equal volumes of nanoparticles/medium for 24 h. After incubation, the samples were washed with PBS by centrifugation in Amicon^®^ centrifugal filter units of 50 KDa cutoff (Millipore) 3000× *g*, 4 °C, in cycles of 10 min.

Sample preparation for LC-MS/MS was done by solubilizing the NLC in 100 mM Tris(hydroxymethyl)aminomethane (Tris) pH 8.5, 1% sodium deoxycholate, 10 mM tris(2-carboxyethyl)phosphine (TCEP), 40 mM chloroacetamide and 1 × complete^TM^ protease inhibitor cocktail (Roche Applied Science, Mannheim, Germany) for 10 min at 95 °C and 1000 rpm (Thermomixer, Eppendorf, Germany). The samples were handled for proteomic analysis according to the solid-phase enhanced sample-preparation (SP3) protocol from Hughes et al. [28]. Enzymatic digestion was done by incubating the samples with 2 µg of Trypsin/LysC overnight at 37 °C and 1000 rpm. For protein analysis, 500 nanograms of each sample was introduced in a nanoLC-MS/MS system composed of an Ultimate 3000 liquid chromatography system attached to a Q-Exactive Hybrid Quadrupole-Orbitrap mass spectrometer (Thermo Scientific, Bremen, Germany). Each sample was loaded onto a trapping cartridge (Acclaim PepMap C18 100Å, 5 mm × 300 μm i.d., 160,454, Thermo Fisher Scientific, Waltham, MA, USA) in a mobile phase of 2% acetonitrile and 0.1% formic acid at 10 μL/min and processed as described in Melo et al. [25]. Protein quantification was conducted by label-free quantification (LFQ). Raw data were processed using Proteome Discoverer 2.5 software (Thermo Scientific, Waltham, MA, USA) and searched against the Uniprot *Bos taurus* database. Proteins with two or more identified unique peptides were considered for protein analysis.

#### 2.4.2. Effect of Protein Corona on NLC Bactericidal Activity

To test the effect of the protein corona on NLC bactericidal activity, NLC60 were prepared and incubated in different media as described in Section 2.4.1. *H. pylori* (grown as described in Section 2.2.1) concentration was adjusted to approximately 1 × 10^7^ CFU/mL in PBS. Different NLC60 concentrations (10^11^, 10^12^, 10^13^ particles/mL) were incubated with the bacteria for 6 h at 37 °C, in microaerophilic conditions. Afterward, samples were collected and plated as described in Section 2.2.2. The plates were incubated at 37 °C for 4–5 days in microaerophilic conditions and the number of viable bacteria/mL was calculated by CFU counting.

### 2.5. Statistical Analysis

Statistical analysis was performed using Graph Pad Prism 8.0 (Graph-Pad Software, San Diego, CA, USA). Data were expressed as mean ± standard deviation. Statistical significance was assessed using Two-way ANOVA followed by Tukey’s multiple comparisons test. Statistically significant differences were considered for *p* < 0.05.

## 3. Results

### 3.1. NLC Optimization

#### 3.1.1. NLC with Different Surface Charge

**Figure 1:**
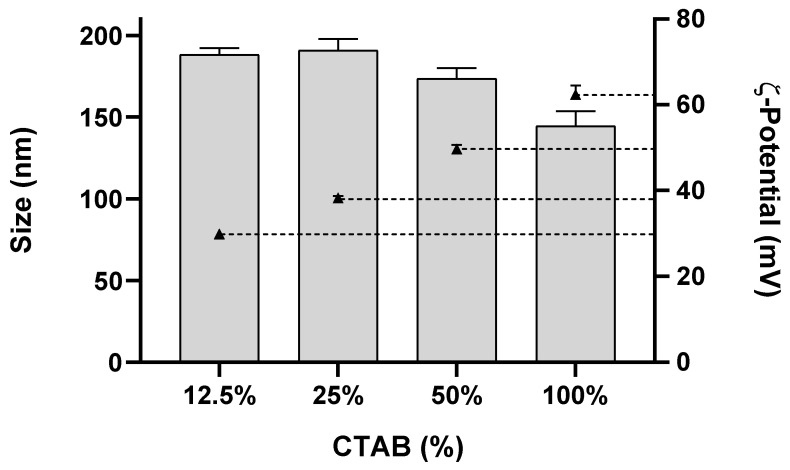
Effect of CTAB/Tween^®^60 ratios on NLC size and ζ-potential. Size was determined using dynamic light scattering (DLS) and ζ-potential using electrophoretic light scattering (ELS). Data are expressed as means ± standard deviation. Bars represent NLC size (average diameter), and triangles with dotted lines represent ζ-potential values: 30 ± 0.3, 38 ± 0.4, 50 ± 0.9 and 62 ± 2 mV.

Blank NLC previously developed by us had an average diameter of 211 ± 8 nm and negative surface charge (ζ-potential close to −28 mV) [19,20]. These NLC were produced with Precirol ATO5^®^, Miglyol-812^®^ and Tween^®^60 using an amplitude of sonication of 60% for 5 min. For positively charged NLC, the same lipids and sonication parameters were used but a cationic surfactant (CTAB) was added. In the optimization process, CTAB was mixed with non-ionic Tween^®^60 in different ratios to access the effect on nanoparticle charge (ζ-potential) and hydrodynamic diameter (Figure 1).

NLC composed of 100% CTAB had a mean size of 148 ± 7 nm and a surface charge of +62 ± 2 mV (Figure 1). Reducing the CTAB percentage (increasing Tween^®^60), increased NLC size (from 148 ± 7 to 188 ± 4 nm), while the surface charge diminished (from +62 ± 2 to +30 ± 0.3 mV). NLC with 12.5% CTAB (NLC CTAB) were selected for further assays since this combination yielded NLC with a similar size and absolute ζ-potential (around |30|mV) to the blank NLC.

#### 3.1.2. NLC with Different Sizes and Surfactants

**Figure 2:**
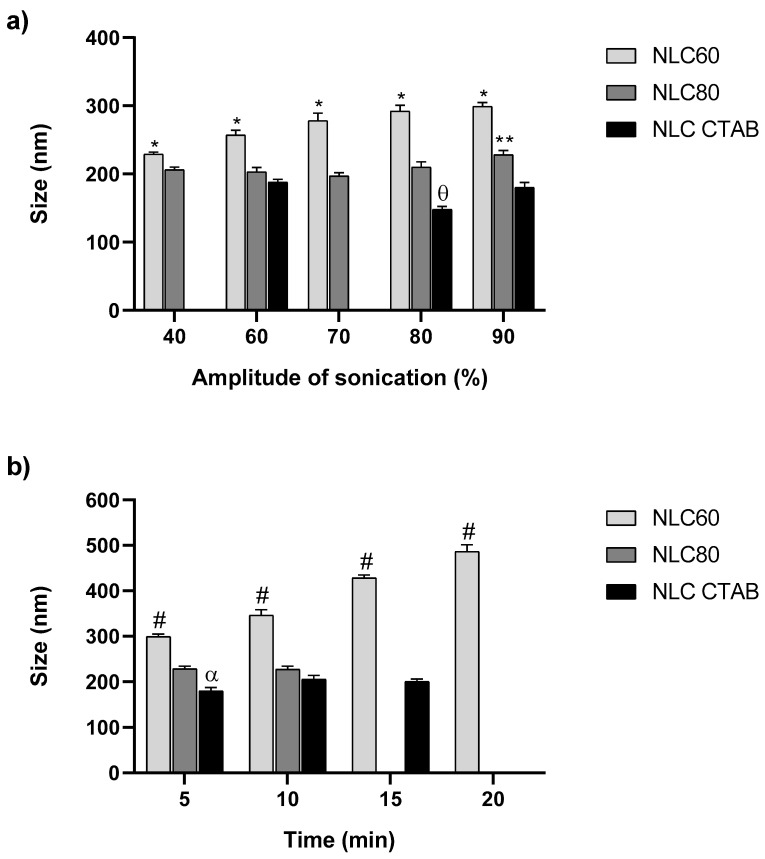
Effect of sonication parameters on NLC size (NLC60, NLC80 and NLC CTAB). (**a**) effect of changing the amplitude of sonication with fixed time (5 min); (**b**) effect of changing the sonication time at fixed amplitude (90%). Size distribution was measured by dynamic light scattering (DLS). Data are expressed as means ± standard deviation. In (**b**), only NLC60 were tested at 20 min, since no alterations were observed for NLC80 and NLC CTAB after 10 and 15 min, respectively. Statistical analysis was performed for each NLC formulation individually using Tukey’s multiple comparisons test. (**a**) * NLC60 size (*p* < 0.05); ** NLC80 size (*p* < 0.05); ϴ NLC CTAB size (*p* < 0.0001); (**b**) # NLC60 size (*p* < 0.0001) and α NLC CTAB size (*p* < 0.05).

The effect of the sonication parameters (amplitude and time) on NLC size was tested in NLC formulations prepared with different surfactants: Tween^®^60 (NLC60), Tween^®^80 (NLC80) and 12.5% CTAB (NLC CTAB) (Figure 2).

The effect of the amplitude of sonication on NLC size (5 min sonication time) varied according to the NLC formulation (Figure 2a). NLC60 had the highest size variation with increasing amplitude of sonication. NLC60 size increased from 230 ± 3 to 300 ± 6 nm when the amplitude of sonication rose from 40 to 90%, respectively. NLC80 had more discrete changes with the increase in amplitude, with size significantly increasing only at 90% of amplitude (varied ≈ 30 nm). NLC CTAB size remained constant independently of the amplitudes tested. Since 90% of amplitude led to higher changes in NLC size, the influence of the sonication time on NLC size was studied at a fixed amplitude of 90% (Figure 2b).

NLC80 and NLC CTAB were not considerably affected by the sonication time: for NLC80 the size remained constant (≈230 nm), while for NLC CTAB a slight increase was observed (180 ± 8 to 205 ± 9 nm) up to 10 min of sonication, plateauing after this (Figure 2b). NLC60 had a higher size variation with different sonication times, increasing up to 486 ± 15 nm at the longest time of sonication (20 min) (Figure 2b).

Overall, only the NLC60 formulation could be modulated by changing the amplitude and time of sonication to create nanoparticles with different sizes. Therefore, NLC60 with three different sizes were obtained: smaller (NLC60_S_), medium (NLC60_M_; control) and larger (NLC60_L_). The conditions selected to prepare these NLC60 are described in Table 2.

#### 3.1.3. NLC for In Vitro Assays

The optimized sonication parameters and the features of all NLC formulations selected for in vitro assays are described in Table 2.

NLC sizes were evaluated by TEM and by DLS. As expected, NLC analyzed by TEM had a smaller size than NLC analyzed by DLS (hydrodynamic size). This difference is associated with the liquid layer that is formed around the nanoparticles when measured in DLS, which is not present when NLC were observed by TEM (dry NLC). However, hydrodynamic sizes are representative in the context of the in vitro assays since the effect of NLC was evaluated in media.

All the NLC had similar, low polydispersity indexes (PdI), with values close to 0.2 (Table 2), which indicates a monodispersed distribution. Regarding the surface charge, NLC60 (independently of size) and NLC80 were anionic, with similar negative ζ-potential (−26 to −30 mV), while NLC CTAB were cationic with a positive ζ-potential close to +38 mV.

**Figure 3:**
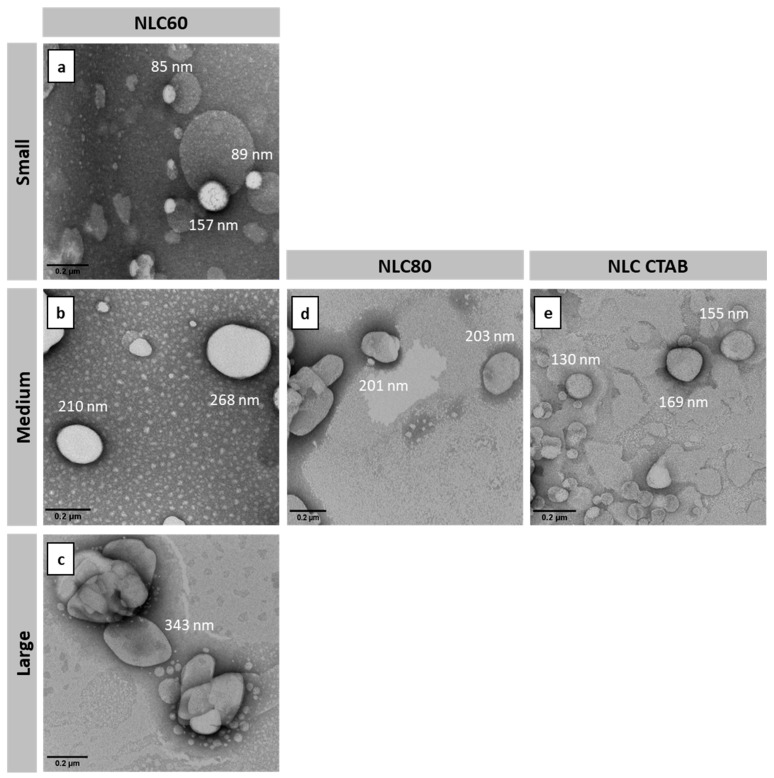
Transmission electron microscopy (TEM) micrographs of the different NLCs. (**a**) NLC60_S_, (**b**) NLC60_M_, (**c**) NLC60_L_, (**d**) NLC80 and (**e**) NLC CTAB. Nanoparticle diameter was measured in triplicate, final size value corresponds to the mean of the measurements. Magnification: 53.000×. Scale bars = 0.2 µm.

TEM assays were also used to evaluate NLC morphology (Figure 3). The images revealed that the produced NLC had a smooth spherical or spheroidal morphology. This spheroidal morphology could be related to the drying process and is more pronounced in larger NLC (Figure 3b–d).

### 3.2. NLC Antibacterial Performance

The antibacterial performance of the NLC formulations (Table 2) was tested against the *H. pylori* J99 strain. Also, their safety towards gut microbiota was assessed by screening against *E. coli* ATCC^®^25922^TM^ and *L. acidophilus*-01, bacteria representative of the normal gut microbiota.

#### 3.2.1. Effect of NLC Charge (Surfactant Composition)

**Figure 4:**
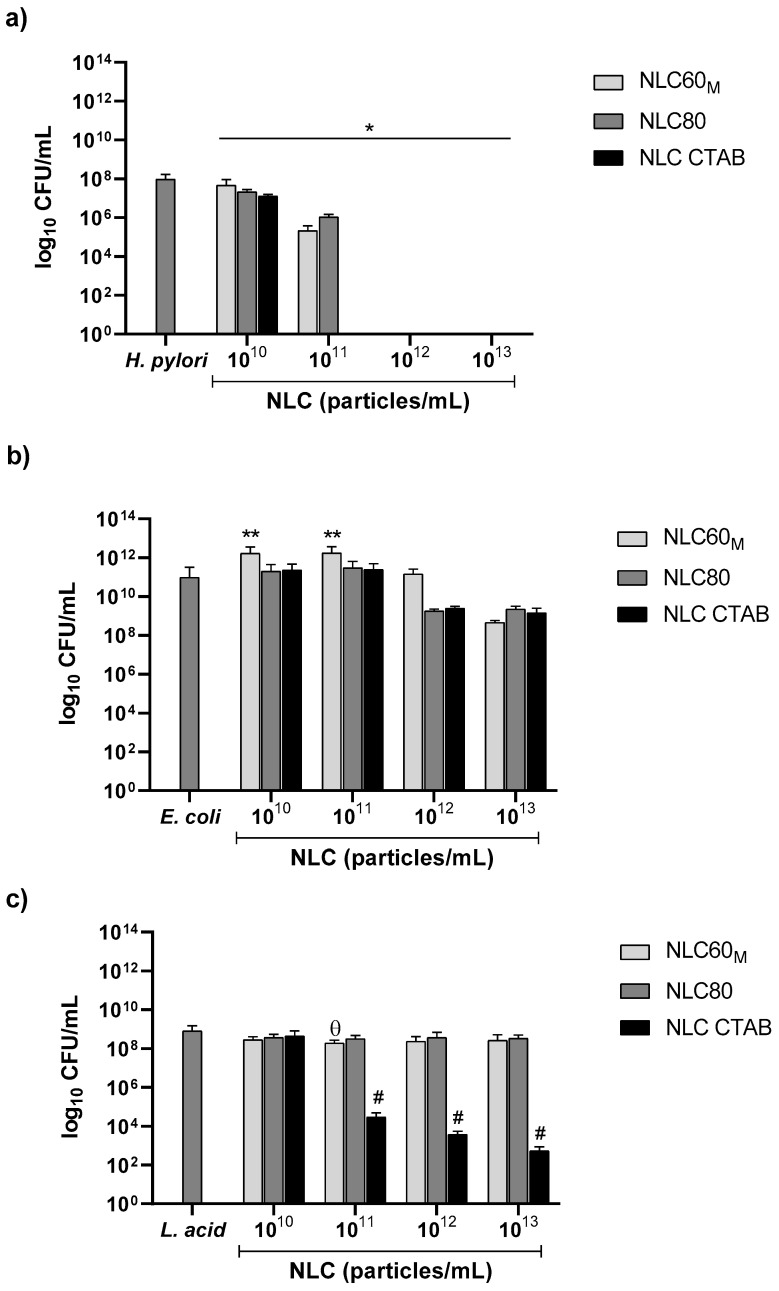
Antibacterial performance of increasing concentrations of NLC60_M_, NLC80 and NLC CTAB against (**a**) *H. pylori* J99, (**b**) *E. coli* ATCC^®^25922™ and (**c**) *L. acidophilus*-01. Assays were performed over 24 h in MHB + 10% FBS medium. Data are expressed as means ± standard deviation. Statistical analysis was performed against the control (bacteria without NLC) using Tukey’s multiple comparisons test. (**a**) * All NLC formulations (*p* < 0.001); (**b**) ** NLC60_M_ (*p* < 0.01); (**c**) ϴ NLC60_M_ (*p* < 0.05) and # NLC CTAB (*p* < 0.05).

NLC with different ζ-potential and surfactant compositions but similar sizes (NLC60_M_, NLC80 and NLC CTAB) were tested against *H. pylori* J99, *E. coli* ATCC^®^25922™ and *L. acidophilus*-01 (Figure 4).

As shown in Figure 4a, all NLC formulations were bactericidal against *H. pylori*. NLC CTAB (positively charged) had the highest bactericidal effect, with complete eradication at 10^11^ particles/mL, whereas NLC60 and NLC80 only achieved the same effect at 10^12^ particles/mL. Moreover, no statistically significant differences were found between NLC60 and NLC80 formulations.

For *E. coli* ATCC^®^25922™ (Figure 4b) and *L. acidophilus*-01 (Figure 4c), no bactericidal activity was observed for either NLC60 or NLC80 at the range of concentrations tested. However, NLC CTAB were bactericidal at 10^11^ particles/mL against *L. acidophilus*-01 (Figure 4c). Thus, despite presenting the highest bactericidal effect against *H. pylori* J99, the cationic NLC CTAB emerged as non-gut microbiota friendly, due to its bactericidal effect on *L. acidophilus*-01.

#### 3.2.2. Effect of Size

**Figure 5:**
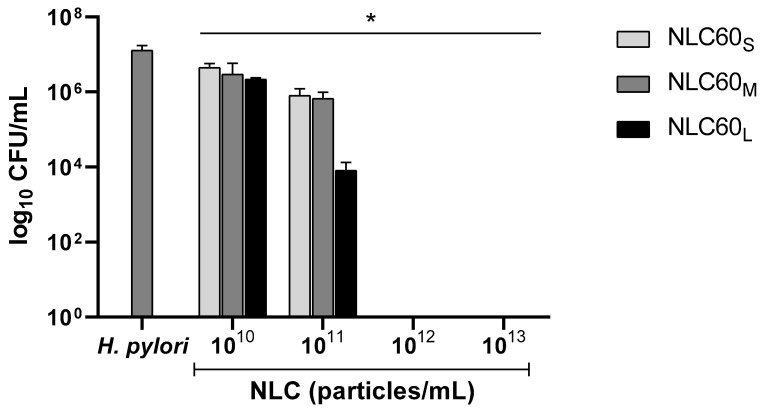
Antibacterial performance of increasing concentrations of NLC60 with different sizes against *H. pylori* J99. NLC60_S_ (147 ± 10 nm), NLC60_M_ (263 ± 8 nm) and NLC60_L_ (443 ± 11 nm). Assays were performed over 24 h in MHB + 10% FBS medium. Data are expressed as means ± standard deviation. Statistical analysis was performed against the control (bacteria without NLC) using Tukey’s multiple comparisons test. * All samples were statistically significantly different from control (*p* < 0.0001).

Different NLC60 sizes (NLC60_S_, NLC60_M_ and NLC60_L_) were tested against *H. pylori* J99 (Figure 5).

Results demonstrated that size influenced NLC60 activity against *H. pylori* (Figure 5). NLC60_L_ (443 ± 11 nm) achieved a bactericidal effect at 10^11^ particles/mL, while NLC60_S_ and NLC60_M_ required 10^12^ particles/mL. Still, at this concentration, all formulations reached full *H. pylori* clearance.

#### 3.2.3. Effect on Different *H. pylori* Morphologies

To assess the NLC bactericidal effect on different *H. pylori* morphologies, NLC60_M_ were tested against bacteria of rod and coccoid morphology. To achieve coccoid morphology, before the bactericidal assay, *H. pylori* was grown for 72 h instead of the usual 16–18 h to promote bacterial stress by nutrient depletion, consequently leading to the change in morphology. The morphological change was confirmed by optical microscopy.

**Figure 6:**
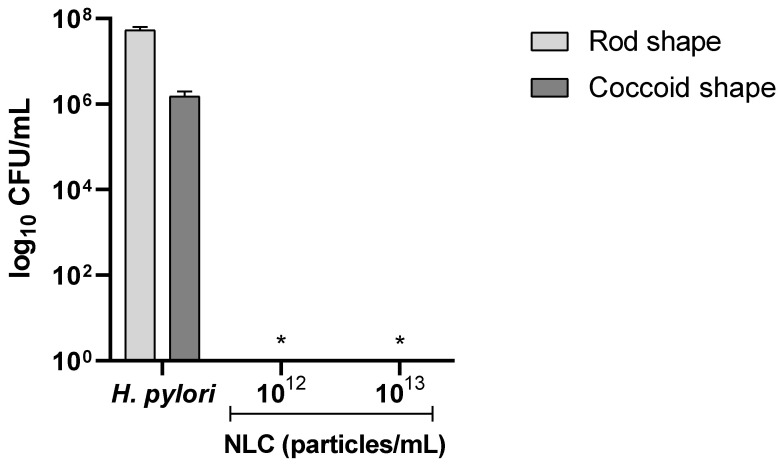
Antibacterial performance of increasing concentrations of NLC60_M_ against *H. pylori* J99 in rod and coccoid morphologies. Assay was performed in PBS over 6 h of incubation. Data are expressed as means ± standard deviation. Statistical analysis was performed against the control (bacteria without NLC) using Tukey’s multiple comparisons test. * Both morphologies (*p* < 0.0001).

Since complete *H. pylori* eradication was observed after 24 h incubation with NLC60_M_ at a concentration of 10^12^ particles/mL and above in the previous assays, the concentrations 10^12^ and 10^13^ particles/mL were chosen to test the effect of NLC60_M_ on both *H. pylori* morphologies after 6 h in PBS. The results are shown in Figure 6.

*H. pylori* was grown in rod- and coccoid-shape and both initial cultures were adjusted to start at 1 × 10^7^ CFU/mL. Since the coccoid-shaped *H. pylori* have a slower metabolism, the bacterium grew less than the rod-shaped *H. pylori* [16]. As expected, a difference in growth was observed in the controls for both morphologies, with rod-shaped bacteria growing about 1.5 logs CFU/mL more than the coccoid-shaped *H. pylori*. Nonetheless, it was observed that NLC60_M_ were effective at the same concentration (10^12^ particle/mL) for both morphologies (Figure 6).

**Figure 7:**
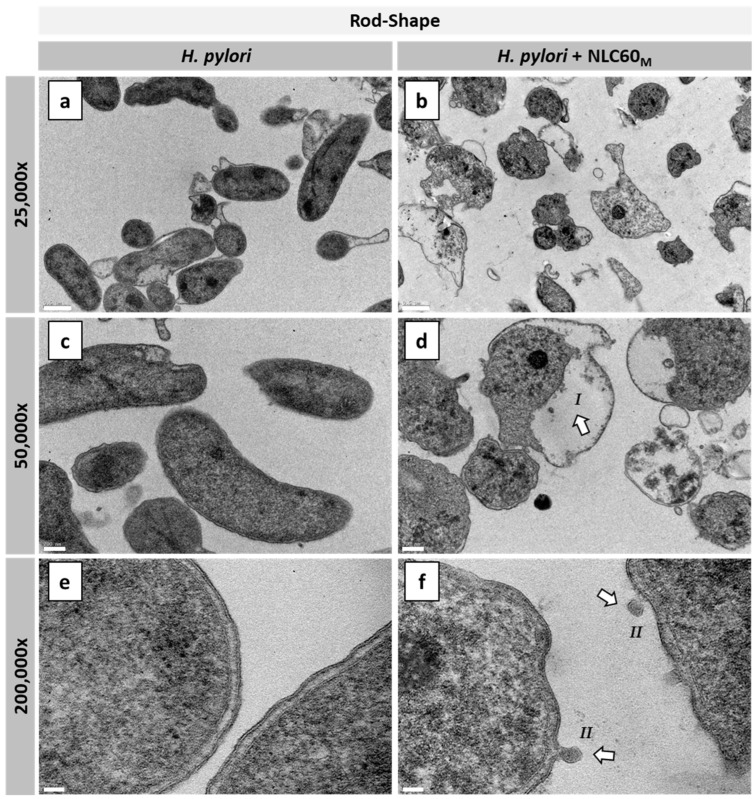

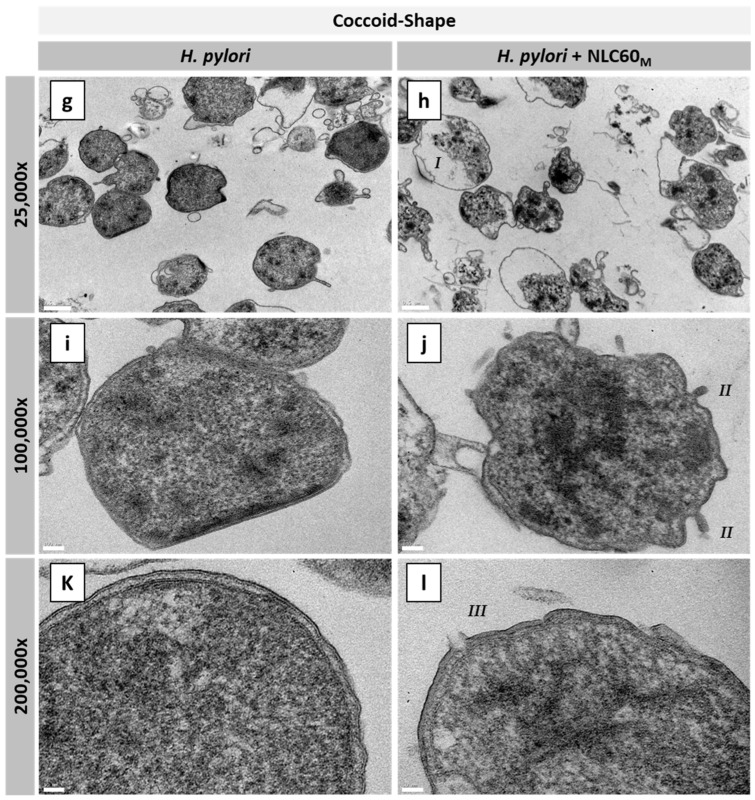
Transmission electron microscopy (TEM) micrographs of rod- and coccoid-shaped *H. pylori* J99 after 6 h of exposure to NLC60_M_ in PBS: (**a**,**c**,**e**) rod-shaped *H. pylori* control, (**b**,**d**,**f**) rod-shaped *H. pylori* exposed to NLC60_M_ (10^12^ particles/mL), (**g**,**i**,**k**) coccoid-shaped control and (**h**,**j**,**l**) coccoid-shaped exposed to NLC60_M_ (10^12^ particles/mL). Scale bars: (**a**,**b**,**g**,**h**) 500 nm; (**c**,**d**) 200nm; (**i**,**j**) 100 nm and (**e**,**f**,**k**,**l**) 50 nm. Arrows indicate: *I*—alterations in the cytoplasm; *II*—vesicle formation and *III*—membrane disruption.

The effect of NLC60_M_ on the *H. pylori* membrane in both morphologies was also studied by transmission electron microscopy (TEM). Figure 7 shows the TEM images of rod- and coccoid-shaped *H. pylori* with (*H. pylori* + NLC60_M_) and without (*H. pylori*) exposure to NLC60_M_ for 6 h in PBS.

TEM images showed that the rod-shaped control bacteria (*H. pylori*) (Figure 7a,c,e) had a healthy morphology, presenting intact cell membranes. Moreover, images of the coccoid-shaped *H. pylori* (Figure 7g,i,k), although in coccoid-shape, also show intact cell membranes. When exposed to NLC60_M_ (Figure 7b,d,f,h,j,l) changes in the cytoplasm (*I*) and cell membrane irregularities, such as the formation of vesicles (*II*) and membrane disruption (*III*), were observed in both morphologies. These results clearly show the effectiveness of NLC60_M_ against both rod and coccoid-shaped bacteria, explaining the bactericidal effect described in Figure 6.

#### 3.2.4. Effect of FBS on NLC Antibacterial Performance against *E. coli* and *L. acidophilus*

**Figure 8:**
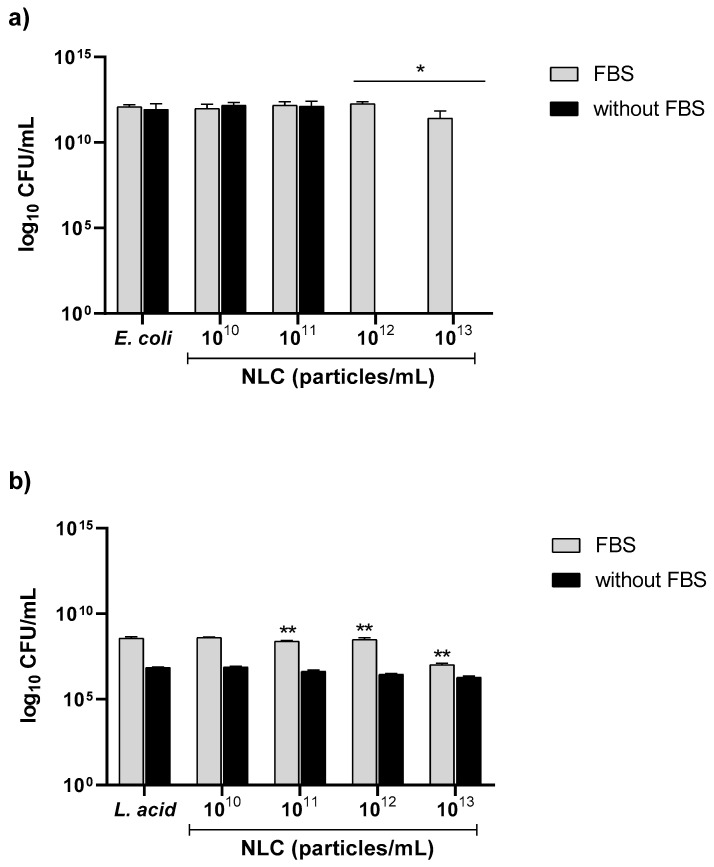
Antibacterial performance of NLC60_M_ against (**a**) *E. coli* and (**b**) *L. acidophilus*-01 grown in MHB with and without 10% FBS after 24 h of incubation. Data are expressed as means ± standard deviation. Statistical analysis was performed against the control medium using Tukey’s multiple comparisons test. * Samples with and without FBS (*p* ˂ 0.05) and ** Samples in FBS (*p* ˂ 0.05).

*H. pylori* is a fastidious bacterium that is auxotrophic for cholesterol [29]. In the antibacterial assays, the medium is supplemented with FBS as a cholesterol source. However, *E. coli* and *L. acidophilus* do not require cholesterol. Therefore, to assess if FBS influences NLC performance, NLC60_M_ were incubated with *E. coli* and *L. acidophilus*-01 in MHB medium with and without FBS (Figure 8).

NLC60_M_ had a bactericidal effect against *E. coli* when FBS was removed from the medium and complete eradication was reached at 10^12^ particles/mL (Figure 8a). The differences in NLC60 activity observed in *E. coli* suggest that the proteins of the FBS used for the in vitro bacterial assays influence NLC activity.

For *L. acidophilus*-01, a difference in growth was observed when comparing the controls of the different media. With FBS in the medium, the bacterium grows 2 logs CFU/mL more than without FBS. However, when exposed to the NLC, no differences were observed either with or without FBS, since NLC60_M_ was not bactericidal (Figure 8b).

### 3.3. NLC Protein Corona

The presence of FBS in the in vitro assay medium affected the NLC activity towards *E. coli*, demonstrating that media composition impacts NLC activity. This could be due to the interaction of proteins present in the medium with the nanoparticles, leading to the formation of a protein corona.

#### 3.3.1. Characterization of NLC Protein Corona

**Figure 9:**
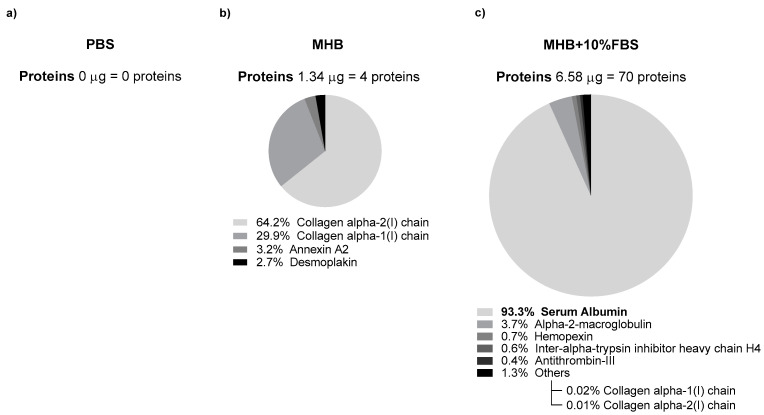
Schematic representation of the adsorbed proteins to NLC60_M_ surface. Protein corona composition in NLC incubated in: (**a**) PBS, (**b**) MHB and (**c**) MHB + 10% FBS. Most abundant proteins represented in respective percentages of total protein adsorption. A total of 500 ng of each sample were analyzed.

Protein corona in NLC60_M_ was evaluated after the incubation of nanoparticles in PBS, MHB and MHB supplemented with 10% FBS. After being washed to remove any potential medium debris and to assure that only the adsorbed proteins would be present in the NLC, the nanoparticles were analyzed by LC-MS/MS (Figure 9).

According to the mass spectrometry results (Figure 9), NLC incubated in PBS had no adsorbed proteins on their surface, as was expected. For NLC incubated in MHB and MHB + 10% FBS, 4 and 70 different proteins were found, respectively, and 2 of them (Collagen alpha 1 and 2) were common to both samples. In MHB + 10% FBS, the predominant protein in the sample was serum albumin with an abundance of 93%. These results demonstrate that NLC60 adsorb proteins on their surface creating a protein corona, the composition of which depends on the media.

#### 3.3.2. Effect of NLC Protein Corona on Antibacterial Performance against *H. pylori*

**Figure 10:**
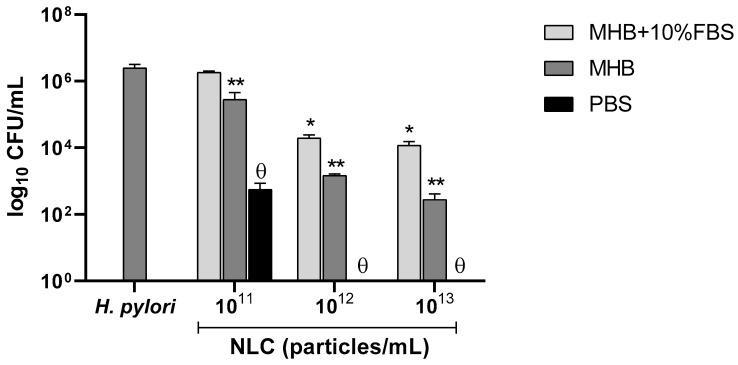
Antibacterial performance of increasing concentrations of NLC60_M_ pre-immersed in PBS, medium (MHB) and medium with FBS (MHB + 10% FBS) against *H. pylori* J99 (6 h in PBS). Data are expressed as means ± standard deviation. Statistical analysis was performed against the control (bacteria without NLCs) using Tukey’s multiple comparisons test. * NLCs incubated in MHB + 10% FBS (*p* ˂ 0.01); ** NLCs incubated in MHB (*p* ˂ 0.0001) and ϴ NLCs incubated in PBS (*p* ˂ 0.0001).

The presence of a protein corona composed of proteins present in the FBS was validated. So, the possibility that this protein corona impacts the NLC antibacterial activity against *H. pylori* was considered. To study the influence of the different media in NLC60_M_ activity towards *H. pylori*, the nanoparticles were pre-immersed in different media as described in Section 2.4.1. The results are shown in Figure 10.

After 6 h of incubation with NLC60_M_ exposed to MHB + 10% FBS, no bactericidal effect was reached in any of the tested concentrations (Figure 10). Non-supplemented MHB showed bactericidal activity at 10^13^ particles/mL, but complete clearance was not obtained. In PBS, NLC60_M_ achieved complete *H. pylori* clearance at 10^12^ particles/mL. Thus, it was observed that the NLC protein corona masks the nanoparticles and delays the bactericidal activity. Additionally, when comparing these results with the MS analysis (Figure 9), it is observed that an increase in protein adsorption to the NLC surface directly correlates with an increasing delay in NLC activity.

### 3.4. Discussion

*H. pylori* is classified by the International Agency for Research on Cancer (IARC) as a group 1 carcinogen [5]. Eleven infectious pathogens have this classification, and, among them, *H. pylori* ranks first as an infectious cause of cancer worldwide [5]. Due to the rise of *H. pylori* resistance to available antibiotics, it is urgent to find alternative solutions to fight this bacterium. Previously, we described that blank NLC per se have bactericidal activity against *H. pylori,* even if organized in biofilms [19,20,24]. Here, the effect of charge, size and protein corona on NLC antimicrobial specificity toward *H. pylori* was explored.

Since the NLC previously described prepared with Tween^®^60 (NLC60) were anionic (−28 mV) [19,20], this surfactant was substituted by the cationic surfactant CTAB to produce cationic nanoparticles (NLC CTAB). NLC prepared with 100% CTAB generated nanoparticles with +62 mV of ζ-potential, but this value was not comparable with the negatively charged NLC60 (ζ-potential −28 mV). Optimization using different CTAB/Tween^®^60 ratios demonstrated a decrease in NLC ζ-potential from +62 mV to +30 mV when the CTAB percentage was changed from 100% to 12.5% (Figure 1). Formulations with two or more surfactants usually have smaller sizes because of the surfactant combination effect [30]. However, the NLC CTAB size increased (from ~150 to ~200 nm) with the decrease in CTAB % (from 100 to 12.5%), which could be explained by the high difference in the molecular weight (MW) of the two surfactants (MW CTAB = 364.45 g/mol and MW Tween^®^60 = 1311.7 g/mol).

Both NLC formulations, the anionic NLC60 and the cationic NLC CTAB were bactericidal against *H. pylori* since a reduction of more than 3 logs CFUs after 24 h incubation was attained (Figure 4a). As expected, the cationic NLC CTAB had a higher bactericidal effect, with total *H. pylori* clearance reached at a concentration 10 times lower (10^11^ particles/mL) than the one used for NLC60 (10^12^ particles/mL). However, despite this promising performance, NLC CTAB were also bactericidal against *L. acidophilus* (one of the bacteria tested as representative of gut microbiota) at a similar concentration (10^11^ particles/mL) demonstrating that they are not selective to *H. pylori* (Figure 4c). This is in accordance with what is described in the literature, since positively charged nanoparticles usually have higher antibacterial activity due to their interaction with the negatively charged bacterial membrane, both in Gram-positive and Gram-negative bacteria [31]. However, NLC CTAB did not affect *E. coli* (Figure 4b). This might be due to the stronger outer membrane of *E. coli* that acts as a selective physical barrier, protecting the bacterium from external threats [32,33].

NLC were also produced using Tween^®^80 (NLC80) since, unlike Tween^®^60, soluble Tween^®^80 has a bactericidal effect against *H. pylori* [24,34,35]. Moreover, it was also reported that Tween^®^80 improved *H. pylori* eradication in infected patients when combined with the usually prescribed antibiotics [35]. The different antimicrobial effects observed between Tween^®^60 (polyethylene glycol (20) sorbitan monostearate; C_64_H_126_O_26_) and Tween^®^80 (polyethylene glycol (20) sorbitan monooleate; C_64_H_124_O_26_) was not clear, since both surfactants are non-ionic with similar MW and structure [36]. This antimicrobial effect might be associated with the unsaturated aliphatic chain of Tween^®^80. However, our results demonstrated no advantages in *H. pylori* bactericidal activity and selectivity for NLC80 compared to NLC60 (Figure 4a). This may be related to the orientation of the surfactant on the NLC surface, since the aliphatic chains are facing the lipid content, exposing its similar polyethylene glycol (20) chain to the aqueous phase.

NLC60 with different sizes (NLC60_S_~150 nm, NLC60_M_~260 nm and NLC60_L_~450 nm) were successfully obtained by the alteration of sonication parameters (time and amplitude). The size of NLC60 increased with an increase in sonication amplitude and time (Figure 2). This was not observed in NLC prepared with the other surfactants, where alterations in sonication parameters did not significantly affect the size of NLC80 and NLC CTAB (Figure 2). The size of NLC CTAB was only changed by altering the ratio of CTAB/Tween^®^60 (Figure 1). Therefore, the effect of size on NLC selective bactericidal activity against *H. pylori* was evaluated using NLC60 (NLC60_S_ NLC60_M_ and NLC60_L_). All NLC60 sizes were bactericidal against *H. pylori* after 24 h incubation, with complete *H. pylori* clearance at 10^12^ particles/mL (Figure 5). At the same time, improved bactericidal performance was observed with NLC60_L_, since the bactericidal effect was reached at a lower NLC concentration (10^11^ particles/mL). This result contradicts the usual conception that smaller nanoparticles have a higher bactericidal effect [37,38,39,40]. However, in most cases, the nanoparticles are designed as drug delivery systems, where the bactericidal effect is intrinsically associated with the drug and not with the nanoparticle per se. Moreover, in the case of metallic nanoparticles, their small size allows them to cross the bacterial membrane causing changes in the bacterial membrane and metabolism, which ultimately promotes bacterial death [38,39,40]. Since these NLC affect *H. pylori* membranes by contact [19], it would be expected that larger NLC, by having a larger contact area with the bacterial membrane, were more efficient.

To have relevance as a new therapeutic strategy against *H. pylori*, ideally, these NLC need to be effective against both *H. pylori* morphologies, since *H. pylori* in a coccoid morphology are more resistant to antibiotics [14]. Until now, few antibiotic-free nanoparticles showed an effect against the coccoid morphology, and the ones that did had compounds incorporated into the nanoparticles, such as linolenic acid into liposomes [41]. NLC60_M_ were bactericidal for both rod- and coccoid-shaped bacteria, achieving complete *H. pylori* eradication at 10^12^ particles/mL (Figure 6). Transmission electron microscopy (TEM) images clearly showed the effect of NLC60_M_ on both morphologies of *H. pylori*, where NLC60_M_ induced the formation of protrusions, vesicles and, in some cases, membrane disruption (Figure 7f,j,l). The release of the cytoplasm was confirmed by observation of free space inside *H. pylori* cells exposed to NLC (Figure 7d,h). However, no significant increase in periplasmic space was observed when compared with the control samples (Figure 7e,f,k,l). These observations corroborate the hypothesis that blank NLC60 destabilize the *H. pylori* bacterial membrane, although why this occurs is still not fully understood [20,24]. *H. pylori* is adapted to cross the gastric mucus layer, which contains highly hydrophilic regions, to reach the epithelial cell layer. As such, it can be speculated that, since these NLC are coated with a very hydrated surfactant (due to the exposed polyethylene glycol), they can be perceived by *H. pylori* as the gastric mucus layer. The unexpected high *H. pylori* adhesion to a polyethylene glycol (PEG) surface was already demonstrated by us [42]. This was not anticipated since PEG surfaces are well known for their “non-fouling” properties, being able to avoid protein adsorption and bacteria adhesion [43].

Therefore, high protein adsorption to NLC60 was not expected due to their hydrated coating (Tween^®^60). However, since bactericidal assays were performed in media supplemented with 10% FBS, the adsorbed protein layer, normally designated by protein corona [44,45,46], was analyzed by mass spectrometry. Protein corona can influence the characteristics of the nanoparticles (e.g., size and charge) as well as their activity, degradation and recognition by the immune system in physiologic conditions and in in vitro settings [47,48]. Results demonstrated that when NLC60_M_ were incubated for 24 h with MHB supplemented with 10% FBS, 6.58 μg of protein (13.2 ng of protein/ng of NLC) were observed, corresponding to the presence of 70 different proteins (Figure 9). Among these proteins, serum albumin was the most prevalent, with an abundance of 93% (6.14 μg of the total 6.58 μg of detected protein). These results are corroborated by FBS composition, which has serum albumin as a major constituent (60 to 67% of total protein composition) [49,50], and by what is documented relatively to protein corona from lipid nanoparticles [51,52,53]. For NLC60_M_ preincubated in MHB, 1.34 μg of protein (2.7 ng of protein/ng NLC) was detected due to the presence of dour proteins, where 94% are collagen (Figure 9). As expected, no protein content was detected when NLC60_M_ were incubated in PBS.

Although only *H. pylori* require FBS in the culture media, FBS was maintained in the selectivity assays, using *E. coli* and *L. acidophilus*, to keep the in vitro assays comparable. In the presence of FBS (NLC with protein corona) NLC60_M_ did not affect *E. coli* and *L. acidophilus* (Figure 8). However, without FBS in the media, NLC60_M_ was bactericidal against *E. coli,* reaching complete clearance at 10^12^ particles/mL (same concentration as for *H. pylori*) (Figure 8a). This was not observed for *L. acidophilus*. Thus, the protein corona masks NLC60_M_ activity against *E. coli.*, being responsible for NLC60_M_ selectivity.

Studies in PBS using NLC60_M_ pre-coated with PBS, MHB and MHB + 10% FBS demonstrated that the protein corona (93% albumin) only delayed the NLC bactericidal effect against *H. pylori* (Figure 10). Nevertheless, this NLC protein corona observed in the in vitro assays would not correspond to an in vivo protein corona, which is a more dynamic process [51,52,53].

### 3.5. Conclusions

NLC with different sizes and charges were successfully developed. All the NLC formulations were effective against *H. pylori*, with the cationic NLC CTAB being 10 times more efficient than the anionic NLC60 and NLC80. However, NLC CTAB was not selective to *H. pylori*, having a similar effect against *L. acidophilus.* A protein corona composed of 93% albumin only delayed NLC60 bactericidal activity against *H. pylori* but hindered their bactericidal activity against *E. coli*. These NLC achieved complete *H. pylori* clearance in both morphologies (rod- and coccoid-shape). These new insights establish NLC60_M_ as bactericidal against the more resistant morphology of *H. pylori* and reveal the possible role of the protein corona in their in vitro selectivity towards *H. pylori*. Overall, these results sustain NLC as a promising antibiotic-free treatment against *H. pylori*.

## Figures and Tables

**Table 1:** Composition of the different nanostructured lipid carriers (NLC).

NLC Formulation						
Lipids				Surfactants		
Code	Milli-Q^®^ Water (mL)	Precirol^®^ATO5 (mg)	Miglyol^®^812 (mg)	Tween^®^60 (mg)	Tween^®^80 (mg)	CTAB (mg)
NCL60_1	4.2			60.0	-	-
NLC60_2	5.0			60.0	-	-
NLC80_1	4.2			-	60.5	-
NLC CTAB_1	4.2	200	90	-	-	17
NLC CTAB_2	4.2			30.0	-	8.5
NLC CTAB_3	4.2			45.0	-	4.3
NLC CTAB_4	4.2			52.5	-	2.2

**Table 2:** NLC formulations chosen for the in vitro assays with different bacteria. Final optimized sonication parameters and characterization by dynamic light scattering (DLS) and electrophoretic light scattering (ELS). Data are expressed as means ± standard deviation.

		Sonication Parameters		Characterization			
				TEM	DLS		ELS
NLC	Formulation (Table 1)	Amplitude (%)	Time (min)	Dry Diameter (nm)	Hydrodynamic Diameter (nm)	PdI	ζ-Potential (mV)
NLC60_S_	NLC60_2	40	5	102 ± 21	147 ± 10	0.25 ± 0.04	−28 ± 0.9
NLC60_M_	NLC60_1	60	5	202 ± 59	263 ± 8	0.20 ± 0.01	−27 ± 0.4
NLC60_L_	NLC60_1	90	20	342 ± 82	443 ± 11	0.23 ± 0.02	−26 ± 0.8
NLC80	NLC80_1	90	5	195 ± 41	237 ± 8	0.22 ± 0.02	−30 ± 0.7
NLC CTAB	NLC CTAB_4	90	10	189 ± 45	211 ± 6	0.24 ± 0.01	+38 ± 0.2

## Data Availability

Upon request, data will be provided.

## Supplementary Materials

The following supporting information can be downloaded at: https://www.mdpi.com/article/10.3390/pharmaceutics14122745/s1, Table S1. Nanostructured lipid carriers (NLC) optimization and characterization. Hydrodynamic diameter determined by dynamic light scattering (DLS) and ζ-potential by electrophoretic light scattering (ELS). Data are expressed as means ± standard deviation.
