# Supplementary material for: How Charge, Size and Protein Corona Modulate the Specific Activity of Nanostructured Lipid Carriers (NLC) against Helicobacter pylori
Source: Pharmaceutics. 2022 Dec 8;14(12):2745. doi: 10.3390/pharmaceutics14122745 (PMC9785867; doi:10.3390/pharmaceutics14122745)
Supplement: Supplementary file 1 [file pharmaceutics-14-02745-s001.zip › pharmaceutics-2036824-supplementary.pdf]

Table S1. Nanostructured lipid carriers (NLC) optimization and characterization. Hydrodynamic diameter determined by dynamic light scattering (DLS) and ζ-potential by electrophoretic light scattering (ELS). Data is expressed as mean ± standard deviation.

	**Sonication Parameters**		**Characterization**		
**Formulation Code**	**Amplitude (%)**	**Time (min)**	**Hydrodynamic diameter (nm)**	**Polydispersity index**	**ζ-Potential (mV)**
NLC60_1	40	5	230 ± 3	0.35 ± 0.02	-25 ± 0.4
NLC60_1	60	5	257 ± 7	0.21 ± 0.02	-26 ± 0.6
NLC60_1	70	5	278 ± 11	0.17 ± 0.03	-27 ± 0.4
NLC60_1	80	5	292 ± 9	0.19 ± 0.01	-29 ± 0.4
NLC60_1	90	5	299 ± 6	0.17 ± 0.02	-28 ± 0.4
NLC60_1	90	10	346 ± 12	0.18 ± 0.01	-24 ± 0.4
NLC60_1	90	15	428 ± 7	0.21 ± 0.02	-24 ± 0.3
NLC60_1	90	20	486 ± 15	0.24 ± 0.02	-27 ± 0.4
NLC60_2	40	5	200 ± 8	0.20 ± 0.01	-29 ± 0.5
NLC80_1	40	5	206 ± 4	0.18 ± 0.03	-29 ± 0.3
NLC80_1	60	5	203 ± 7	0.20 ± 0.01	-27 ± 0.8
NLC80_1	70	5	197 ± 5	0.19 ± 0.01	-22 ± 0.1
NLC80_1	80	5	210 ± 8	0.20 ± 0.02	-25 ± 0.7
NLC80_1	90	5	228 ± 6	0.20 ± 0.01	-27 ± 0.3
NLC80_1	90	10	227 ± 7	0.20 ± 0.02	-25 ± 0.5
NLC CTAB_1	60	5	145 ± 9	0.21 ± 0.02	62 ± 2.1
NLC CTAB_1	60	10	112 ± 5	0.25 ± 0.01	54 ± 1.3
NLC CTAB_1	80	10	107 ± 2	0.26 ± 0.01	57 ± 2.2
NLC CTAB_2	60	5	174 ± 7	0.19 ± 0.01	50 ± 0.9
NLC CTAB_3	60	5	191 ± 7	0.18 ± 0.01	38 ± 0.4
NLC CTAB_4	60	5	188 ± 4	0.20 ± 0.02	30 ± 0.3
NLC CTAB_4	60	10	192 ± 7	0.19 ± 0.01	32 ± 0.2
NLC CTAB_4	80	5	148 ± 4	0.21 ± 0.01	45 ± 0.7
NLC CTAB_4	90	5	180 ± 8	0.20 ± 0.01	38 ± 0.4
NLC CTAB_4	90	10	205 ± 9	0.20 ± 0.01	29 ± 0.7
NLC CTAB_4	90	15	200 ± 6	0.20 ± 0.01	33 ± 0.5

Note: The values on this table may not exactly correspond to the ones presented in the main paper due to batch-to-batch variation.
